# It’s time to put plastics on the health security agenda: a call to action for Indonesia and Australia

**DOI:** 10.1136/bmjgh-2025-023316

**Published:** 2026-08-10

**Authors:** Sekar Aqila Salsabilla, Andy Fefta Wijaya, Fadillah Amin, Sujarwoto Sujarwoto, Laura Downey

**Affiliations:** 1 Department of Public Administration, Universitas Brawijaya, Malang, East Java, Indonesia; 2 The George Institute for Global Health, UNSW Sydney, Sydney, New South Wales, Australia; 3 The George Institute for Global Health, Imperial College London, London, England, UK

**Keywords:** Global Health, Health policies and all other topics

**Summary Box**

- Indonesia has become the primary recipient of Australia’s exported plastic waste, adding to more than 7 million tonnes of domestic plastic waste generated each year.
- A significant proportion of this waste is openly burned or poorly managed, releasing toxic pollutants linked to respiratory disease, cardiovascular stress, adverse pregnancy outcomes and elevated cancer risk—firmly placing plastic waste as an emerging public health threat and not just an environmental issue.
- Community-driven, multisectoral interventions targeted towards addressing this issue demonstrate local capacity to mitigate the harmful impacts of plastic waste; however, the potential efficacy of such interventions is undercut by the continued influx of international waste imports.
- Recognising plastics as a regional issue and integrating plastic waste management into Comprehensive Strategic Partnerships (CSP), such as the recently signed Australia–Indonesia CSP, could enhance regional environmental security, reduce health risks and support pan-national achievement of sustainable development objectives and health protection goals.

The global escalation of plastic waste has reached a critical level, with plastic use projected to nearly triple from 460 million tonnes (Mt) in 2019 to 1231 Mt by 2060, and environmental leakage expected to double to 44 Mt annually.^1^ This trajectory poses serious ecological and public health risks, particularly as emerging evidence shows that microplastics can cross the placental barrier, exposing fetuses to contamination even before birth.^2^ From the moment infants are born they inhale air already contaminated with hazardous plastic-derived pollutants which accumulate over time across the life course, exacerbating the risks of several health concerns including respiratory disease and infection, cardiovascular disease, infertility and cancer. We’re setting our children up to suffer and they deserve better.

## Indonesia has become a dumping ground for the world’s plastic waste

Indonesia faces an increasingly severe waste management crisis, receiving more than 200 000 tonnes of imported waste,^3^ effectively positioning the country as a new dumping ground for plastic waste from Australia, the USA and several European nations.^3^ In 2024, more than 25 Mt of waste remain unmanaged in 2024.^4^ Evidence indicates that much of the imported material ultimately enters the Indonesian environment, where it is either openly burned or routed to informal burning facilities as an energy source for cooking and small-scale household industries,^5^ resulting in acute and potentially dangerous exposure to fine particulate matter (PM2·5), airborne particles with an aerodynamic diameter of 2·5 µm or less that penetrate deep into the lungs, while even smaller plastic-derived particles can infiltrate the bloodstream,^6^ thereby amplifying health and environmental risks.

Against this backdrop of environmental exposure, emerging evidence suggests that community perceptions of plastic burning are shaped less by abstract environmental concerns and more by everyday trade-offs between perceived risks and immediate practical needs. Participants in our research programme conducted across six villages in Malang Regency, East Java frequently acknowledged visible pollution and short-term associated symptoms such as throat irritation or coughing during and immediately after a burning event, indicating an awareness that burning plastic can be harmful. However, these issues were often viewed as short-lived and relatively unimportant when weighed against the necessity to manage plastic waste efficiently in the absence of viable alternative waste management methods.^7^

These intersecting environmental exposures and social practices carry significant public health implications. Chronic exposure to pollutants released during plastic combustion is associated with increased risks of respiratory disease, certain cancers, cardiovascular dysfunction, impaired lung development in children and adverse pregnancy outcomes.^8 9^ Vulnerable populations—including children, older adults and individuals with pre-existing health conditions—face disproportionate risks. Routine exposure in affected communities represents a cumulative health burden. Beyond direct physiological effects, pollution-related illness can exacerbate socioeconomic vulnerability through lost productivity and increased healthcare expenditures, placing further strain on those least able to manage it.^10^ Taken together, these effects underscore that mitigating plastic waste burning is not only an environmental priority, but also a critical public health concern and a matter of social justice and equity.

## Communities are at the forefront of fighting the plastic waste issue

Our research programme, where community members, in partnership with a diverse range of policy, healthcare, environment, academic and civil society stakeholders, are actively engaged in co-producing solutions, demonstrates that the plastic waste crisis can be addressed through collective, community-driven action.^11^ These efforts are supported by a local government that is equally committed to advancing collective progress in plastic waste management through community-based solid waste initiatives such as waste banks, community education, financial incentives for environmental workers and programmes that position women at the forefront of empowered action. This focus on women is central, aligning with one of the 10 priority programmes of women-led organisations in Indonesia that focus on environmental sustainability, and reflects the pivotal role women play in household waste management, as they are primarily responsible for decisions related to plastic consumption and disposal. While the deployment of our initiative is relatively new and we await formal evaluation evidence on health and environmental outcomes, our early process evaluation findings indicate a shift in behaviour and community norms.^12^ As one community member and study participant noted,

I used to burn waste, but the intervention showed me it can be sorted and reused – what seemed useless before now has value.

### Local efforts to mitigate plastic waste are being undercut by international waste importation

To date, efforts to manage plastic waste in Indonesia have been largely unable to address the problem effectively, as substantial volumes of waste continue to be imported both legally and illegally into the country—undermining the local community’s capacity to mitigate the crisis. Although Indonesian regulations allow the importation of recyclable materials with up to 2% impurity,^13^ illegal imports of plastic and paper scrap with extremely high contamination levels have resulted in large accumulations of waste that cannot be processed domestically, as much of the material consists of non-recyclable plastic packaging, including multilayer films unsuitable for local recycling systems. Most plastics waste imports to Indonesia come from Australia—in 2023–2024 alone, Australia exported 22 333 tonnes of plastic waste to Indonesia,^14^ further exacerbating the nation’s solid waste burden.

These pressures are compounded by weak enforcement of existing waste importation laws, fragmented regulatory oversight and insufficient sorting and processing infrastructure, which allow contaminated or low-value materials to enter domestic waste streams despite formal restrictions.^15^ The resulting accumulation and informal disposal of unusable waste increases environmental contamination and associated community health risks.

### Leveraging the Australia–Indonesia CSP for health security: an opportunity for change

**Figure 1:**
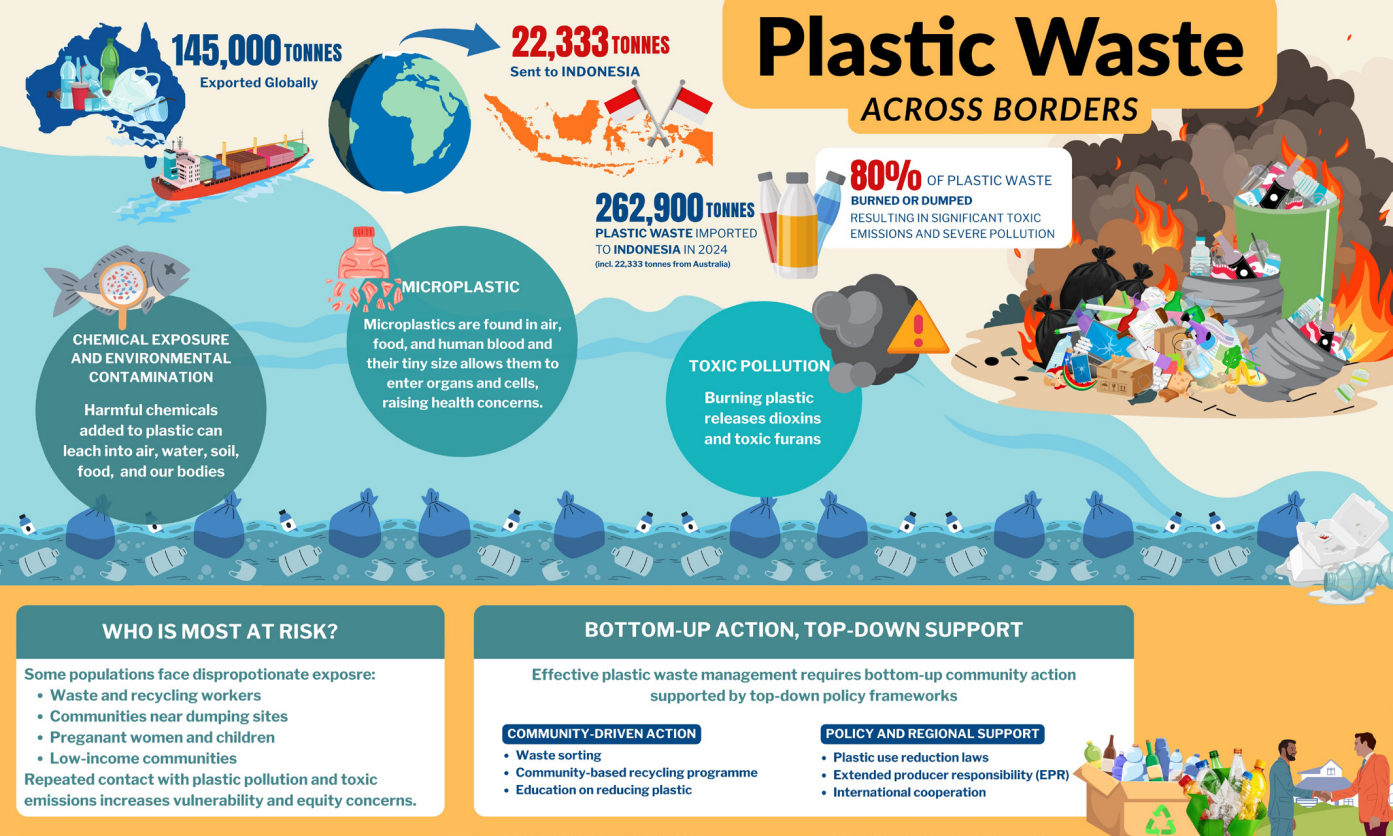
Plastic waste across borders.

Tackling the plastic waste burden requires inter-country coordination and collective effort. The recently signed Comprehensive Strategic Partnership (CSP) between Australia and Indonesia emphasises shared interests in economic development, political and security collaboration, maritime cooperation and broader societal exchange, highlighted recently by the Australian prime minister as a ‘new era’ in relations between the neighbouring countries.^16^ Given the cross-boundary nature of plastic pollution, there is a pressing need to elevate plastics as a regional priority within the CSP framework. Plastics generate harms across their life cycle—from toxic emissions during production and open burning to pervasive microplastic exposure—many of which cross national borders (figure 1). Airborne pollutants, contaminated food systems and marine debris circulate regionally, exposing populations in both countries and beyond to preventable risks with implications for environmental integrity, human health and economic and social well-being. Framing plastic pollution as a shared health security concern aligns directly with CSP provisions to strengthen regional prevention, preparedness and response to health threats, as well as cooperation between public health institutions and knowledge exchange networks. It also complements development priorities focused on climate resilience and sustainable growth. Embedding plastics within these established areas of cooperation underscores that unmanaged waste is not merely an environmental issue, but a driver of long-term public health pressures and social vulnerability—making coordinated action a logical extension of existing bilateral commitments.

For Australia, inadequate regional plastic governance is not a distant problem but a direct concern. Transboundary pollution threatens marine ecosystems, food security and coastal livelihoods, while unmanaged waste contributes to long-term public health pressures. Coordinated action—including curbing illegal plastic waste trade and improving cross-border governance—would strengthen joint capacity between two countries to reduce exposure, prevent harm and promote sustainable development.

Positioning plastic waste management as a central element of the CSP strategic dialogue aligns environmental protection with regional well-being, generating shared health, environmental and economic benefits for both nations. Addressing plastic waste through this lens would strengthen regional collaboration to reduce pollution leakage, mitigate environmental and public health risks, and support the growth of domestic recycling and waste-processing industries. Embedding plastics within bilateral commitments also advances broader sustainable development goals, reinforcing environmentally responsible growth, regional resilience and long-term ecological security.

## Conclusions

Plastic pollution represents a pressing global challenge that demands urgent, coordinated action to avert severe environmental degradation and public health risks. The inequitable distribution of plastic waste, exacerbated by cross-border export and import of plastic shipments, undermines local mitigation efforts such as that led by our multidisciplinary team, and highlights the transboundary nature of the problem. Strategic partnerships, such as the CSP between Australia and Indonesia, provide a critical platform to elevate health and environmental security within the regional agenda. By placing plastics at the forefront of multilateral dialogue and cooperation, these partnerships can drive meaningful enforcement, promote sustainable waste management and ensure that collective efforts yield tangible benefits for both ecological integrity and human well-being across the region.

## Data Availability

There are no data in this work.
